# The emerging role of IG-IMRT for palliative radiotherapy: a single-institution experience

**DOI:** 10.3747/co.v16i3.304

**Published:** 2009-05

**Authors:** R. Samant, L. Gerig, L. Montgomery, R. MacRae, G. Fox, B. Nyiri, K. Carty, M. MacPherson

**Keywords:** Image-guided radiotherapy, intensity-modulated radiotherapy, palliation

## Abstract

Many modern radiotherapy centers now have image-guided intensity-modulated radiotherapy (ig-imrt) tools available for clinical use, and the technique offers many options for patients requiring palliative radiotherapy. We describe a single-institution experience with ig-imrt for short-course palliative radiotherapy, highlighting the unique situations in which the technique can be most effectively used.

## 1. INTRODUCTION

Radiotherapy, an essential part of the management of cancer patients, is used in both the curative and the palliative setting^1^,^2^. Significant advances have been made in technology since radiation was first used to treat cancers more than a century ago^3^. The most dramatic advances have occurred since the end of the 1980s: New imaging modalities for treatment planning have included computed tomography (ct), magnetic resonance imaging, and positron-emission tomography; and sophisticated planning approaches such as intensity-modulated radiation therapy (imrt) 3–5 are possible. New image-guided (ig) and adaptive radiotherapy techniques are also emerging as feasible approaches to make radiotherapy more precise and effective^6^,^7^. However, much of the literature for these modern, sophisticated techniques involves high-dose radiotherapy with curative intent^5^,^6^,^8^–^11^.

Much less has been published about utilizing these advanced technologies in the setting of short-course palliative radiotherapy^8^,^12^. In fact, it is often noted that relatively simple techniques are already very effective^13^,^14^, and one of the priorities has been to get treatment started quickly. Because days to weeks may be required to attain the full symptom improvement benefits from palliative radiotherapy, it makes sense to use whichever approach can be effective, can minimize side effects, and can be planned relatively quickly. Delaying the start of radiotherapy because of a complicated treatment planning process does not make sense. For patients requiring palliative radiotherapy, specialized clinics have even been developed where treatment (with one or a few fractions of radiotherapy) is planned and administered on the same day that the consultation takes place^15^. Usually, fluoroscopic simulation and simple treatment approaches such as direct fields or parallel opposed fields are used in these clinics. However, are we utilizing the available radiotherapy scanning, planning, and treatment tools to the fullest extent possible in the palliative setting?

We believe that, in certain situations, it is possible to use state-of-the-art ig-imrt approaches to deliver palliative radiotherapy in a safe, efficient, and effective manner without excessive wait times. It seems intuitive that, if radiotherapy could be focused more precisely, then it could be more effective and should lead to fewer side effects, because the tumour can be targeted more accurately and normal structures can be avoided. In the palliative setting, in which the goal tends to be delivery of low-to-moderate doses of radiation with minimal toxicity^13^, an ig-imrt approach would seem to be most appropriate. Here, we review our experience using ig-imrt on our helical Tomo-Therapy unit (TomoTherapy Incorporated, Madison, WI, U.S.A.) in the palliative setting.

## 2. PATIENTS AND METHODS

In the spring of 2005, the Ottawa Hospital Cancer Centre installed a helical TomoTherapy unit (htu), and after a commissioning process that took 2 weeks, it started treating patients in September 2005. The htu was acquired mainly for research purposes, and many of the patients were on study protocols. However, having quickly realized the value and versatility of an ig-imrt approach, we began using it for selected palliative situations in which standard approaches might not be safe, and for unique situations in which palliative radiotherapy, although effective, would be difficult to administer. We subsequently created a protocol for scanning, planning, and treating in one setting patients who require urgent palliative radiotherapy for symptom relief^16^. Here, we describe our experience using ig-imrt in the setting of short-course palliative radiotherapy with the htu during the first 2 years of full operation. The intent of treatment—as well as the treatment details, including dose and fractionation—was recorded electronically. The data were collected in an Excel spreadsheet (Microsoft Corporation, Redmond, WA, U.S.A.) and subsequently analyzed.

## 3. RESULTS

In 2005, just 25 patients were treated on the htu as the staff became familiar with the equipment and the new processes involved in planning and treatment. However, during the 2-year period from January 2006 to December 2007, a total of 227 patients were treated and, in 57 cases (25%), the intent was listed as palliative. Symptomatic bone and brain metastases were the most common indications for treatment (40% and 19% respectively). Approximately 90% of these palliative radiotherapy courses consisted of 10 or fewer fractions, and the median dose was 2000 cGy in 5 fractions over 1 week. The number of patients undergoing palliative radiotherapy with ig-imrt increased from 19 in 2006 to 38 in 2007. Those numbers represented approximately 0.8% and 1.7%, during 2006 and 2007 respectively, of the total palliative radiotherapy courses given at our centre (almost half of the total radiotherapy courses administered annually are for palliation).

**FIGURE 1:**
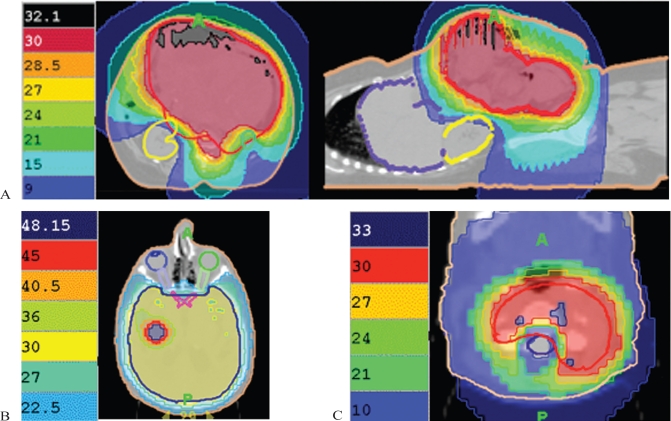
(A) Distribution for large recurrent colon cancer being treated with abdominopelvic radiation with sparing of the liver and right kidney. (B) Whole-brain radiation with concurrent boost to area of gross recurrence. (C) Re-irradiation of vertebral body with relative sparing of spinal cord.

We have used ig-imrt for palliation in several types of scenarios, as outlined in the next few paragraphs. Initially, patients with complicated and symptomatic metastatic or locally advanced disease were so treated, because treatment with conventional approaches would not allow delivery of adequate radiation doses to the planning target volume (ptv) in combination with sparing of normal tissues. Figure 1 shows examples, including dosimetry, of particularly useful indications for ig-imrt.

The first type of situation [Figure 1(A)] involves tumour volumes that are very large and adjacent to normal structures. Here, the tumour can be treated with considerable normal-tissue sparing because, with image guidance, small margins (0.5–1.0 cm) are feasible, and imrt allows for “dose painting,” in which adequate ptv coverage is combined with avoidance of large volumes of normal tissue. We have used this approach for bulky abdominopelvic recurrence of colorectal carcinoma and for extensive mesothelioma involving the thorax and abdomen. In these types of cases, acute toxicity is quite manageable and not prohibitive, even with hypofractionated palliative radiotherapy regimens (2000 cGy in 5 fractions over 1 week, or 3000 cGy in 10 fractions over 2 weeks).

The second type of situation [Figure 1(B)] involves cases in which we can boost certain areas of the ptv to higher doses. Traditionally, we would use two phases, but with ig-imrt, we can perform a concurrent boost with very good dose gradients. This technique is not more time-consuming in terms of planning or more resource-intensive than is a conventional approach, but it is far more convenient for patients. For example, we believe that it is well suited for limited brain metastases, in which we can deliver the standard dose of 3000 cGy in 10 fractions over 2 weeks to the whole brain and simultaneously boost the areas of gross disease to a further 10–15 Gy. We have found that the planning for this treatment is simpler than performing whole-brain irradiation followed by a stereotactic boost.

The third type of situation [Figure 1(C)] deals with re-irradiation, especially when critical structures such as the spinal cord are involved. Here, we use immobilization, very small margins, and stringent dose–volume constraints to treat the tumour and to obtain maximal normal-tissue sparing. This approach has been used at our centre for re-treating painful bony vertebral metastases where the dose received by the spinal cord must be limited. We have been able to re-treat vertebrae to 3000 cGy in 10 fractions over 2 weeks, with the cord getting only 30%–50% of the prescribed dose (which would keep exposure levels well within acceptable tolerances).

Finally, we also piloted a protocol in which patients undergo megavoltage ct image acquisition, target delineation, imrt treatment planning, and verification of position before treatment, and finally delivery of the first fraction of radiation all in one visit (full details published elsewhere^16^). Subsequently, we also developed a protocol called Tomo-pal (TomoTherapy—Planning and Administration Linked), during which we scan, plan, and deliver single-fraction treatment all in one session for patients requiring urgent radiotherapy. The approach is similar to that used in our rapid palliative radiotherapy clinic, designed for rapid access to single-fraction treatment especially suited for treating bone metastases. The key advantage of Tomo-pal is that we are able to scan, plan, and treat patients in approximately 1 hour, which is extremely convenient for patients and also has the potential to reduce toxicity (both acute and late).

**FIGURE 2:**
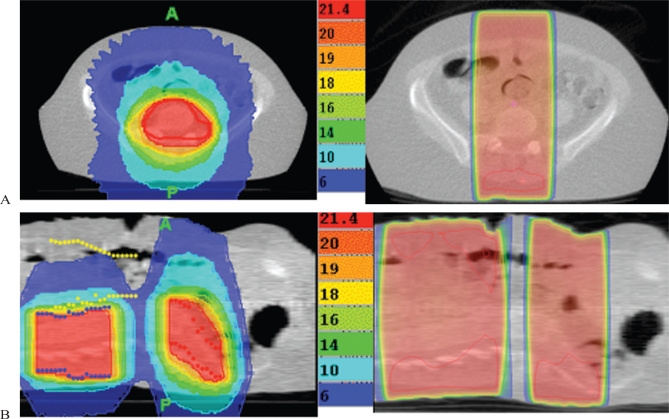
*(A) Treatment of sacrum with image-guided intensity-modulated radiation therapy* (*ig-imrt*) *as compared with traditional fields, showing significant reduction of high-dose region and hotspots. (B) Treatment of two adjacent sites of vertebral body metastases with* *ig-imrt* *as compared with traditional fields, showing dramatic reduction in radiation dose to gastrointestinal tract with the more sophisticated approach.*

Comparisons of the quick imrt plans with standard dosimetry using simple direct or parallel fields demonstrate much more homogeneous doses to the target volumes, reduced hotspot areas, and much lower doses to adjacent normal tissues (Figure 2). At our centre, with clinical mark-ups or fluoroscopic simulation, we have traditionally used 1.5–2.5 cm margins around areas of disease when delivering palliative radiotherapy. With an ig-imrt approach, we perform volumetric planning to reduce the irradiated volume, and we tend to use smaller margins, ranging from 0.5 cm to 1.0 cm. We have gained confidence in the use of such small margins because of the verification imaging for patient position and target localization before each fraction of radiotherapy is delivered. The delivery quality assurance measurements also show good agreement between planned and delivered dose. The imrt plans also tend to be better than our three-dimensional treatment plans because of the ability to tailor the radiotherapy to various unusual tumour geometries and because of the smaller margins that can be used. Given the limited follow-up thus far, no significant complications have been noted in the short term in our treated patients. The approach discussed here has been used most often for painful bone metastases, including single-fraction treatment.

## 4. DISCUSSION

Palliative radiotherapy forms a very large proportion of the workload at cancer centres^13^,^17^,^18^, and our data are consistent with those findings. However, palliative radiotherapy usually does not require the same proportion of resources^13^–^15^,^19^, largely because even simple palliative radiotherapy techniques can be very effective and lead to high rates of symptom improvement^13^,^14^. In certain situations, though, modern radiotherapy technology needs to extend to the palliative setting when appropriate. Studies have already demonstrated that ct-based imaging and planning of palliative radiotherapy is superior to that with conventional approaches that use clinical mark-ups or fluoroscopy^20^–^22^, and using ig-imrt for palliation is a natural extension.

When using ig-imrt, we can more confidently use relatively small margins (0.5–1.0 cm) around the areas of tumour, because patients will be re-imaged for verification of position and tumour location before each treatment. We have been impressed by the reproducibility of patient set-ups and the minimal shifts required. The distributions achieved also deliver a much more homogeneous dose to the ptv and minimize dose to the adjacent normal tissues, even with a rapid imrt planning process.

Based on our experience, the four main scenarios in which we have found ig-imrt to be most useful are these:

- Tumours that are large and that have complex geometric configurations are difficult to treat with conventional approaches, especially when adjacent to critical structures. For this type of treatment, we use a standard planning ct for image acquisition. Fairly complex planning is then performed. Although this planning can be labour-intensive, it can allow for radiotherapy to areas traditionally considered too large or too difficult to treat. This approach is appropriate when patients have symptomatic disease not responding to other approaches.
- A region is being treated, but a certain portion should be boosted to a higher dose. This situation occurs most often with limited brain metastases, in which we can use our ig-imrt equipment to deliver standard whole-brain radiation doses with a concurrent boost to the areas of gross disease. This approach tends to be simpler than whole-brain radiation followed by subsequent stereotactic radiotherapy. The planning is no more difficult, and the approach is obviously more convenient for patients.
- Re-irradiation to certain areas to treat the tumour (which likely responded well to previous radiotherapy) and the dominant area of symptomatic progression. In this scenario, minimizing radiotherapy dose to structures such as the spinal cord is important. Reports have been published about the value of imrt for spinal and paraspinal tumours^11^,^12^. Again, a standard planning ct is required, as is complex imrt planning, but we have not found this work to be much more onerous than complex planning using a three-dimensional conformal approach.
- In our streamlined process, ig-imrt has proved extremely useful for scanning, planning, and treating patients all in one session. In this approach, all procedures can be carried out safely and efficiently with the patient remaining on the treatment couch in one room during the entire time. These cases—for example, painful bone metastases—usually have less complicated planning, but the treatment distributions are still far superior to those that can be achieved using standard approaches. And we can achieve these superior results in the same time, or less, than it would take to treat the patient with simpler radiotherapy techniques. Multiple targets can also be effectively treated using this approach.

Our experience suggests that the scope of palliative radiotherapy will expand in the future if we start to embrace and utilize these ig-imrt technologies in the appropriate settings. In situations in which we may previously have thought that the size of the tumour, its anatomic location, or its geometry precludes the use of radiotherapy, imrt has the potential to change that thinking, especially if image guidance, which can reduce margins around the tumour and radiotherapy dose adjacent normal tissues, is also incorporated. The ig-imrt technique also offers more opportunities for safe re-irradiation to previously treated sites. And in many situations, ig-imrt can be as fast and efficient as simpler approaches, yet the delivery of the radiotherapy will be more conformal. With experience, imrt treatment planning can be streamlined and carried out relatively quickly for many common situations. We have also been able to redesign some our processes to make sure that patients requiring urgent palliative radiotherapy can be treated just as rapidly with ig-imrt as they can be with a Cobalt-60 unit or a linear accelerator.

Our results indicate mainly the feasibility of ig-imrt for palliative situations; however, clinical results with more relevant patient outcomes should be forthcoming as more centres embrace this approach. But there is no reason to believe that this approach cannot be at least as effective as traditional palliative radiotherapy treatment, in which clinically meaningful symptom improvement is documented in 60%–80% of patients^13^,^23^.

So far, we have not had any concerns whatsoever about the safety of the approach, and substantial literature has already been published with regard to the use of imrt in the curative and adjuvant settings. In general, our patients treated palliatively with ig-imrt appear very happy to undergo such an efficient process. We look forward to seeing more cancer centres publishing their results with ig-imrt for palliation.

## 5. CONCLUSIONS

In the palliative setting, ig-imrt is feasible and efficient, and offers a broad range of options for patients with symptomatic cancer not responding to other therapies. Although ig-imrt is certainly not necessary for all patients requiring palliative radiotherapy, in specific situations it may be the most appropriate approach. Its future holds great promise for patients with advanced cancer who could benefit from radiotherapy.
